# Incidence of the 22q11.2 deletion in a large cohort of miscarriage samples

**DOI:** 10.1186/s13039-017-0308-6

**Published:** 2017-03-09

**Authors:** Melissa K. Maisenbacher, Katrina Merrion, Barbara Pettersen, Michael Young, Kiyoung Paik, Sushma Iyengar, Stephanie Kareht, Styrmir Sigurjonsson, Zachary P. Demko, Kimberly A. Martin

**Affiliations:** Natera, Inc., 201 Industrial Road, San Carlos, 94070 CA USA

**Keywords:** 22q11.2 deletion, Products of conception, Miscarriage, Single nucleotide polymorphism (SNP) microarray, Bioinformatics

## Abstract

**Background:**

The 22q11.2 deletion syndrome is the most common microdeletion syndrome in livebirths, but data regarding its incidence in other populations is limited and also include ascertainment bias. This study was designed to determine the incidence of the 22q11.2 deletion in miscarriage samples sent for clinical molecular cytogenetic testing.

**Results:**

Twenty-six thousand one hundred one fresh product of conception (POC) samples were sent to a CLIA- certified, CAP-accredited laboratory from April 2010–-May 2016 for molecular cytogenetic miscarriage testing using a single-nucleotide polymorphism (SNP)-based microarray platform. A retrospective review determined the incidence of the 22q11.2 deletion in this sample set. Fetal results were obtained in 22,451 (86%) cases, of which, 15 (0.07%) had a microdeletion in the 22q11.2 region (incidence, 1/1497). Of those, 12 (80%) cases were found in samples that were normal at the resolution of traditional karyotyping (i.e., had no chromosome abnormalities above 10 Mb in size) and three (20%) cases had additional findings (Trisomy 15, Trisomy 16, XXY). Ten (67%) cases with a 22q11.2 deletion had the common ~3 Mb deletion; the remaining 5 cases had deletions ranging in size from 0.65 to 1.5 Mb. A majority (12/15) of cases had a deletion on the maternally inherited chromosome. No significant relationship between maternal age and presence of a fetal 22q11.2 deletion was observed.

**Conclusions:**

The observed incidence of 1/1497 for the 22q11.2 deletion in miscarriage samples is higher than the reported general population prevalence (1/4000–1/6000). Further research is needed to determine whether the 22q11.2 deletion is a causal factor for miscarriage.

## Background

The 22q11.2 deletion is the most common microdeletion in humans, and is responsible for causing the distinct range of features associated with the 22q11.2 deletion syndrome, which can include congenital heart defects, hypocalcemic hypoparathyroidism, T-cell mediated immune deficiency, palate abnormalities, and intellectual disability [1, 2]. The vast majority of 22q11.2 deletions are *de novo* and are caused by meiotic nonallelic homologous recombination events between low-copy repeats [3]. Although the majority (~90%) of patients share a common 2.0–3.5 Mb deletion, approximately 7% of patients have a smaller 1.5 Mb deletion nested within the common deleted region, and ~3% have rarer atypical deletions occurring outside this region [1, 3].

Population-wide estimates of the frequency of the 22q11.2 deletion have ranged from 1/4000 to 1/6000 [4, 5], although, because none of these studies were prospective, they are subject to both under-ascertainment and referral biases. True population prevalence is therefore believed to be higher [1]. Recent studies performed in prenatal cohorts have indicated a higher prenatal incidence of ≥1/1000 for the 22q11.2 deletion [6–8]. However, given that these studies were retrospective and involved a substantial percentage of cases referred for invasive diagnostic procedures (e.g., due to ultrasound anomalies), these studies are also subject to ascertainment bias. In the absence of a newborn screening program for the detection of 22q11.2 deletion syndrome, true incidence of the deletion remains unknown.

Recognizing the wide difference between prenatal and postnatal estimates of prevalence, we sought to determine the frequency of the 22q11.2 deletion in a large cohort of miscarriage samples using a single-nucleotide polymorphism (SNP)-based genotyping microarray. In addition to identifying aneuploidies, the SNP array-based method can detect subchromosomal imbalances such as the 22q11.2 deletion at a much higher resolution than traditional karyotyping (>0.5 Mb vs. >10 Mb) [9–11]. Although chromosomal microarray (CMA)-based approaches have already become routine in the pediatric setting and are increasingly being used for the testing of prenatal and adult samples [8, 12, 13], the use of CMA as a first-line test for the analysis of products of conception (POC) specimens is a relatively new application of this technology [9, 14, 15].

## Results

**Fig. 1:**
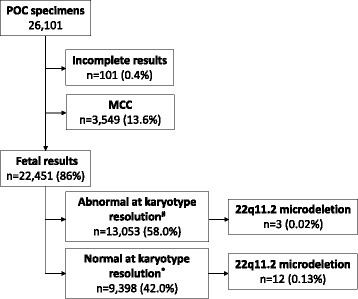
Summary of the study cohort. ^#^Had chromosome abnormalities that could be detected at the resolution of traditional karyotyping (≥10 Mb). ^*^Had no apparent chromosome abnormalities that could be detected at the resolution of traditional karyotyping

Of 26,101 total POC specimens tested, fetal results were obtained for 22,451 (86%) cases. The remaining 3650 (14%) cases were excluded from the analysis due to having maternal cell contamination (MCC) (*n* = 3549, 13.6%) or incomplete results (*n* = 101, 0.4%; Fig. 1). A 22q11.2 deletion was detected in 15 (0.07%) of the 22,451 cases with fetal results, yielding an overall incidence of 1/1497. Two of the 15 cases with deletions were present in a pair of twins; as the twins were dizygotic, we considered the two cases as separate events.

**Fig. 2:**
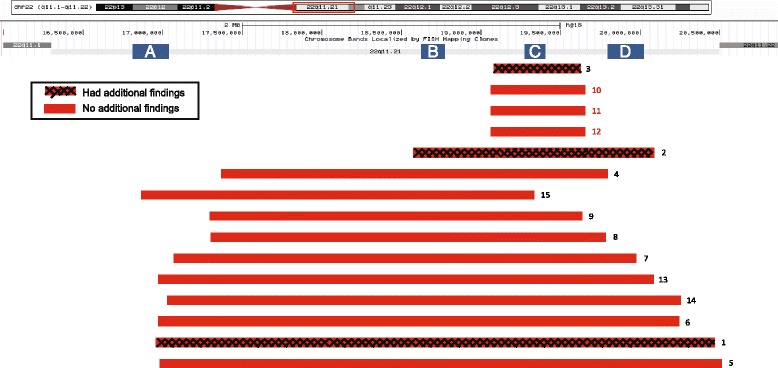
Schematic of each deletion in the 22q11.2 region. Based on coordinates from the NCBI36/hg18 genome browser. The common A–-D low copy repeats are shown in *blue boxes*

Fifty-eight percent (13,053/22,451) of the POC specimens with fetal results were considered abnormal (i.e., had chromosome abnormalities detectable by traditional karyotyping, >10 Mb; Fig. 1). Of these, a 22q11.2 deletion was identified in three cases (incidence, 1/4351), one with a typical 3.5 Mb deletion and a finding of Klinefelter syndrome (47,XXY), one with a nested 1.5 Mb deletion and a finding of trisomy 15, and the third with a rarer 0.65 Mb deletion and a finding of trisomy 16 (Table 1). Among the 42% (9398/22,451) normal cases (i.e., no chromosome abnormalities detectable by traditional karyotyping), a 22q11.2 deletion was identified in 12 cases (incidence, 1/783; Fig. 1). One of these had an additional finding of maternal heterodisomic uniparental disomy of chromosome 17; no other cases had additional findings detectable by the SNP-based array, including other copy number variants (CNVs) (resolution of >0.5 Mb; Table 1). Of the 12 ‘normal’ cases that presented with the 22q11.2 deletion, three cases (two in the set of twins) had 0.72 Mb deletions; the remaining nine cases had the common 2.4–3.5 Mb deletion (Table 1, Fig. 2). Each case is schematically represented against the hg18 browser ideogram for chromosome 22. The common A, B, C, and D low-copy repeats (LCRs) for the 22q11.2 deletion are shown. There is one small region (190 Kb) between LCR-B and LCR-C that is common to all 15 cases (Fig. 2). The only gene in this region is *SCARF2*.

**Table 1:** Description of cases with a 22q11.2 deletion

Case no.	Category^a^	NCBI36/hg18 Genomic Coordinates (GRCh38/hg38 Genomic Coordinates)	Deletion size (Mb)	Parental origin	Additional findings
1	Abnormal	16,930,000–20,430,000 (18,067,234–21,745,711)	3.5	Maternal	XXY
2	Abnormal	18,630,000–20,130,00) (20,262,477–21,445,711)	1.5	Maternal	Trisomy 15
3	Abnormal	19,100,000–19,750,000 (20,415,710–21,065,711)	0.65	Maternal	Trisomy 16
4	Normal	17,400,000–19,780,000 (19,032,487–21,095,711)	2.4	Maternal	Maternal UPD17
5	Normal	16,980,000–20,500,000 (18,117,233–21,815,711)	3.5	Maternal	None
6	Normal	16,940,000–20,250,000 (18,077,234–21,565,711)	3.3	Maternal	None
7	Normal	17,140,000–19,940,000 (18,772,487–21,255,711)	2.8	Maternal	None
8	Normal	17,280,000–19,790,000 (18,912,487–21,105,711)	2.5	Maternal	None
9	Normal	17,280,000–19,700,000 (18,912,487–21,015,711)	2.4	Maternal	None
10^b^	Normal	19,070,000–19,790,000 (20,385,710–21,105,711)	0.72	Maternal	None
11^b^	Normal	19,070,000–19,790,000 (20,385,710–21,105,711)	0.72	Maternal	None
12	Normal	19,070,000–19,780,000 (20,385,710–21,095,711)	0.71	Maternal	None
13	Normal	16,940,000–20,130,000 (18,077,234–21,445,711)	3.2	Paternal	None
14	Normal	17,010,000–20,250,000 (18,147,233–21,565,711)	3.2	Paternal	None
15	Normal	16,890,000–19,290,000 (18,027,234–20,605,713)	2.4	Paternal	None

^a^Based on the presence or absence of chromosome abnormalities that were detectable at the resolution of traditional karyotyping (i.e., ≥10 Mb)

^b^Non-identical twin gestation

Eighty percent (12/15) of the 22q11.2 deletions identified were present on the maternally inherited chromosome (Table 1). Because mothers were not consented for analysis of their samples outside the scope of fetal results, it is unknown how many deletions on the maternal chromosome were *de novo* versus inherited deletions. Furthermore, the status of deletion on the paternal chromosome (cases 13,14 and 15) cannot be determined because paternal samples were not analyzed.

There was no significant difference in mean maternal age (32.6 years; range, 19.0–41.3 years; standard deviation [SD], 5.9) for the 15 cases with the deletion compared with the entire analysis cohort (35.0 year; range, 15.0–52.0 year; SD, 5.3) (*p* = 0.38).

Gestational age information, which was available for 10 of the 15 POC specimens with the 22q11.2 deletion, ranged from 6.4 to 13.6 weeks. Among those, 80% (8/10) of the miscarriages occurred in the first trimester and the remainder occurred early in the second trimester. However, because gestational age at loss was available for less than 20% of the study cohort in total, no inference could be drawn about the relationship between gestational age and presence of the 22q11.2 deletion.

## Discussion

Although gross chromosomal imbalances have been shown to be present in approximately two-thirds of all first trimester miscarriages [9, 16], relatively little information on the frequency of the 22q11.2 deletion in miscarriage is available. To gain insight into this question, we reviewed a large dataset of microarray results from POC specimens for the presence of 22q11.2 deletions. Among 22,451 miscarriage samples with fetal results, we observed the 22q11.2 deletion at an overall incidence of 1/1497, which is significantly more frequent than the reported population prevalence of 1/4000–1/6000 [4, 5]. Possible explanations for this discrepancy include the published general population prevalence potentially being an underestimate, and/or that some affected fetuses have major anomalies that lead to fetal demise [17, 18]. For example, it is known, that cardiac defects are the primary cause [87%] of mortality in infants with the deletion [1]. Consistent with this, other recent studies have found a higher frequency of the 22q11.2 deletion in prenatal cohorts and stillbirths (1/233 to 1/946) [6–8, 13, 19].

The high variability of phenotypic presentation of the 22q11.2 deletion is well known, but it is not fully understood why some individuals with the 22q11.2 deletion are more severely affected than others [20, 21]. Recent evidence suggests a role for additional genetic variants that modify risk for congenital heart defects in some patients with the deletion [22]. Conversely, other studies have found no evidence of an increase in novel genome-wide CNVs in patients with the 22q11.2 deletion [23]. However, there is still the potential for other undetectable mutations and /or environmental factors to impact the severity of cases with the 22q11.2 deletion as was proposed to modify clinical severity of the 16p21.2 deletion and termed a ‘two-hit hypothesis’ [24].

In this study, we found the incidence of 22q11.2 deletions in samples with additional abnormalities was significantly lower than its incidence in samples without them (incidence, 1/4351 vs. 1/783; *p* < 0.01). A previous study comprising a subset of this study’s cohort (the first 2392 samples of 26,101 total) found that the distribution of all subchromosomal CNVs, including the 22q11.2 deletion, was similarly skewed, with a higher proportion of variants present in cytogenetically normal samples [9]. The authors of that study suggested that the higher incidence of copy-number changes in cytogenetically normal samples suggested that these findings “likely contributed to miscarriage causality” [9]. That study also found that the frequency of concurrent double aneuploidies in miscarriage cohorts was lower than was expected based on the reported incidence of each individual anomaly [9]. Presumably more than one significant genetic abnormality leads to selective pressure against implantation, or against continuation of pregnancies beyond 6 weeks. Likewise, if the 22q11.2 deletion were an independent cause of miscarriage, it would likely be observed less frequently in cases with additional chromosomal anomalies.

The combination of the 22q11.2 deletion’s high phenotypic variability, high frequency in stillbirth and prenatal samples and high incidence found in this study suggests that the published general population incidence may be an underestimate and/or that the 22q11.2 deletion could be a causal factor for miscarriage.

Other findings of this study were consistent with previous studies. First, the majority (67%) of 22q11.2 deletions identified in this study had the common LCR-A to LCR-D, 2.0–3.5 Mb-sized, deletion [1]; the remaining deletions ranged in size from 0.65 to 1.5 Mb. Second, 80% (12/15) of 22q11.2 deletions in this study were on the maternally inherited chromosome, similar to previous studies that found a higher proportion of 22q11.2 deletions on the maternal chromosome (76% in prenatally diagnosed cases and 60% in postnatal cases) [17, 23]. Although the exact reason for this skew toward maternal origin is unknown, several factors have been considered, including an increased rate of recombination in this region in female germ cells, a mediating role of the Y chromosome due to homology with the 22q11.2 region, reduced fitness for males with the deletion, and a skewed inheritance pattern in male vs. female offspring [23–25].

Interestingly, a region containing one known gene, *SCARF2,* was common to all fifteen 22q11.2 deletion cases. Mutations in *SCARF2* are associated with Van Den Ende-Gupta syndrome, an autosomal recessive condition characterized by craniofacial and skeletal malformations [26]. *SCARF2* is thought to play a role in cell signaling pathways but it is not well characterized. It may play an important role the development of various organ systems [26].

Approximately 7% of individuals who have a child with a 22q11.2 deletion also have the deletion [1]. Therefore, identification of a 22q11.2 deletion in a miscarriage specimen should prompt evaluation of the parent(s) to determine whether there are clinical and reproductive risks for the parents. Additional medical screening and care is recommended for all individuals with the 22q11.2 deletion, including adults diagnosed later in life [27, 28]. For adults with the deletion, the recurrence risk for each subsequent pregnancy is 50%. Parents with an affected child, but who do not have the deletion themselves, may have a slightly elevated (~1%) recurrence risk in each pregnancy, likely due to gonadal mosaicism [1]. Thus, knowing if a POC specimen has the 22q11.2 deletion can lead to better recurrence risk counseling and future pregnancy management for families.

The results of this study show a higher 22q11.2 deletion incidence in the miscarriage population than literature reports of the live-born population. This implies that either the population prevalence of the 22q11.2 deletion in the live-born population has been underestimated or that the 22q11.2 deletion is a causative factor in miscarriage. Further studies are needed to determine the true incidence of the 22q11.2 deletion syndrome in the live-born and prenatal population. Additionally, the advantages of chromosomal microarray analysis support its use as a first-line test for the analysis of POC samples.

## Methods

A retrospective review of 26,101 consecutive fresh POC specimens received by a single Clinical Laboratory Improvement Act (CLIA)-certified, College of American Pathologists (CAP)-accredited laboratory for clinical miscarriage testing over a 6-year period (April 2010–May 2016) was performed to determine the incidence of the 22q11.2 deletion. A maternal blood sample was requested with each specimen. For each patient, information about maternal age, gestational age, egg donor use, and indication for testing was requested, and all samples were de-identified before review. Additional clinical information including previous pregnancy history, method of conception, pregnancy records and family history was not collected by the laboratory. This study was granted a waiver of the requirement for documentation of informed consent by the institutional review board (E&I ID# 15148-01).

Data on all clinical findings were previously published for the first 2392 cases of this data set, which included one case with the 22q11.2 deletion [9]. Additionally, all syndromic CNVs in karyotypically normal samples from 17,424 cases in this data set were presented at the American Society of Reproductive Medicine 2016 meeting. This included 6 cases with the 22q11.2 deletion [29].

Upon receipt at the commercial reference laboratory, POC specimens were processed by separating chorionic villi from maternal decidua using a standardized technique [30]. These, along with the maternal samples, were genotyped using lllumina CytoSNP-12b microarrays, which measures approximately 300,000 SNPs across the genome (roughly one every 10 kb) according to the manufacturer’s instructions (Illumina, Inc., San Diego, CA). After a genomic sample is run on a SNP array the results must pass an in-house quality control test before further analysis is done. Genotyped samples were analyzed for DNA copy number, uniparental disomy (UPD), parental origin of chromosome abnormalities, and maternal cell contamination (MCC) using the previously described proprietary Parental Support^TM^ algorithm [31]. In short, the allele ratios are calculated for each locus across a chromosome, and the clustering of allele ratios is indicative of the copy number for that chromosome. Comparison of the SNP identities between the maternal and POC data is used to identify maternal cell contamination, the parental origin of aneuploidy and unbalanced chromosome segments. Since parents were not consented for testing of their DNA nor is the father genetic material tested, we are not able to determine whether 22q11.2 deletions are inherited or de novo. Samples were classified as ‘abnormal’ or ‘normal’ based on the presence or absence of chromosome abnormalities that could be detected at the resolution of traditional karyotyping (i.e., >10 Mb). Coordinates for copy number variants (CNVs) >0.5 Mb were entered into the NCBI36/hg18 genome browser to determine clinical significance based on genes affected and previously reported overlapping CNVs. These coordinates were then converted to the GRCh38/hg38 assembly coordinates using the Lift Genome Annotations tool. To determine the statistical significance of maternal age values and of the incidence of the 22q11.2 deletion, independent two-sample t-tests were performed.

## Abbreviations

CNVs: Copy number variants
LCR: Low-copy repeats
Mb: Millions of base pairs
MCC: Maternal cell contamination
POC: Products of conception
SNP: Single-nucleotide polymorphism
UPD: Uniparental disomy
